# Supplementary material for: Urban public space initiatives and health in Africa: A mixed-methods systematic review
Source: PLOS Glob Public Health. 2024 Oct 15;4(10):e0003709. doi: 10.1371/journal.pgph.0003709 (PMC11478912; doi:10.1371/journal.pgph.0003709)
Supplement: S2 Table — (DOCX) [file pgph.0003709.s006.docx]

**Table 2: Systematic review inclusion and exclusion criteria**

| **Domain** | **Inclusion criteria** | **Exclusion criteria** |
| --- | --- | --- |
| Study design | Studies must contain empirical data and primary or secondary analyses of these data. All study designs were considered eligible | Literature reviews, narrative overviews, commentaries, opinion pieces, or any format not providing sufficient information to allow for data extraction |
| Participants | Urban populations in Africa. No age or sex/gender restrictions | Studies focused on contexts outside of Africa or rural settings in Africa |
| Exposures | Public spaces in the urban built or natural environment. This included outdoor spaces, shared neighbourhood spaces such as compounds, green spaces, blue spaces etc | Indoor spaces e.g. buildings or public spaces outside urban areas |
| Comparators | All eligible, if used |  |
| Outcomes | Non-communicable disease risk factor or outcome reduction. This includes:  i) Behaviors – examples include increased physical activity, consumption of healthy diets, improved stress management, social cohesion.  ii) Environmental change – examples include increases in the number of sidewalks, street lighting and signage, air and water quality.  iii) Improvement in access (defined as adequacy, availability, accessibility, affordability, and appropriateness) to services – examples include improved access to health information  iv) Participation of the population – examples include improved social cohesion due to weekly exercise gatherings  v) Improved outcomes – examples include reduction in diabetes, injury, mental illness, cardiovascular diseases, chronic respiratory conditions |  |
| Timing | Studies must have been conducted in African countries and published since 1990 | Studies published before 1990 were excluded from the study |
| Setting | Residents of cities in all African countries as defined by the African Union list of member states | Residents of settings outside of urban Africa |
| Language | All languages considered |  |
