# Supplementary material for: Urban public space initiatives and health in Africa: A mixed-methods systematic review
Source: PLOS Glob Public Health. 2024 Oct 15;4(10):e0003709. doi: 10.1371/journal.pgph.0003709 (PMC11478912; doi:10.1371/journal.pgph.0003709)
Supplement: S3 Table — (DOCX) [file pgph.0003709.s007.docx]

**Table 3: List of Included Countries**

| Algeria | Mali |
| --- | --- |
| Angola | Mauritania |
| Benin | Mauritius |
| Botswana | Mayotte |
| Burkina Faso | Morocco |
| Burundi | Mozambique |
| Cameroon | Namibia |
| Canary Islands | Niger |
| Cape Verde | Nigeria |
| Central African Republic | Republic of the Congo |
| Chad | Réunion |
| Comoros | Rwanda |
| Democratic Republic of the Congo or Zaire | São Tomé and Príncipe |
| Djibouti | Senegal |
| Egypt | Seychelles |
| Equatorial Guinea | Sierra Leone |
| Eritrea | Somalia |
| Ethiopia | South Africa |
| Gabon | South Sudan |
| Gambia | St Helena |
| Ghana | Sudan |
| Guinea | Swaziland |
| Guinea Bissau | Tanzania |
| Ivory Coast or Côte d'Ivoire | Togo |
| Kenya | Tunisia |
| Lesotho | Uganda |
| Liberia | Western Sahara |
| Libya or Jamahiriya | Zimbabwe |
| Madagascar | Zambia |
| Malawi |  |
