# Supplementary material for: Urban public space initiatives and health in Africa: A mixed-methods systematic review
Source: PLOS Glob Public Health. 2024 Oct 15;4(10):e0003709. doi: 10.1371/journal.pgph.0003709 (PMC11478912; doi:10.1371/journal.pgph.0003709)
Supplement: S4 Table — (DOCX) [file pgph.0003709.s008.docx]

**Table 4: List of senior and junior researchers who reviewed and extracted data**

| **Name** | **Designation** |
| --- | --- |
| Ebele R.I. Mogo | Senior researcher |
| Tolu Oni | Senior researcher |
| Louise Foley | Senior researcher |
| Taibat Lawanson | Senior researcher |
| Rose Alani | Senior researcher |
| Felix Assah | Senior researcher |
| Catherine Dominic | Junior researcher |
| Nnenna Onyemaobi | Junior researcher |
| Ayodipupo S. Oguntade | Junior researcher |
| Ghazel Mukhtar | Junior researcher |
| Salimon Muyiolu | Junior researcher |
| Olanike Buraimoh | Junior researcher |
| Doris Seyinde | Junior researcher |
| Okwong Walter | Junior researcher |
| Temitope Ogunjimi | Junior researcher |
| Clarisse Mapa-Tassou | Junior researcher |
| Lia Chatzidiakou | Junior researcher |
| Colin Farr | Junior researcher |
| Stéphanie Teguia | Junior researcher |
| Nfondoh Blanche | Junior researcher |
| Damilola Odekunle | Junior researcher |
| Damilola Olalekan | Junior researcher |
| Chimba Sanga | Junior researcher |
| Crespo’o Ndiabamoh | Junior researcher |
| Richard Unuigboje | Junior researcher |
| Iorse Mvendaga Paul | Junior researcher |
| Victor Onifade | Junior researcher |
| Toluwalope Ogunro | Junior researcher |
| Ife Agboola | Junior researcher |
| Ngwa Edwin | Junior researcher |
