# Supplementary material for: Urban public space initiatives and health in Africa: A mixed-methods systematic review
Source: PLOS Glob Public Health. 2024 Oct 15;4(10):e0003709. doi: 10.1371/journal.pgph.0003709 (PMC11478912; doi:10.1371/journal.pgph.0003709)
Supplement: S5 Table — (DOCX) [file pgph.0003709.s009.docx]

| **Name of Initiative** | **City, Country, Year** | **Summary of the initiative** | **Partners involved** | **Noted outcomes** | **Lessons on implementing public space initiatives in Africa** |
| --- | --- | --- | --- | --- | --- |
| 1. [Changing faces competition](https://www.pps.org/article/communities-in-nairobi-compete-to-contribute-to-public-space) | Nairobi, Kenya  2020  (informal settlement) | Different youth groups competed against each other in creating the cleanest, greenest, and safest public space in the area. It included transforming garbage sites to a community garden and playground | Youth led community-based organizations, county government, Dandora Transformation League, Aga Khan University, UN HABITAT | Increased number of play areas for children and green parks | Lack of funding is the major challenge  Training and community engagement are key to transforming public spaces |
| 1. [The street for all](https://www.codatu.org/actualites/douala-cameroon-great-success-for-the-car-free-day/) | Douala, Cameroon  2019 | A car free day was organized within Doula municipality to improve urban mobility | Mobilize Your City, Doula Urban Community Team | Not reported | Not reported |
| 1. [Model Street](https://world-habitat.org/world-habitat-awards/winners-and-finalists/making-cities-together/) | Nairobi, Kenya  2020  (informal settlement) | Gaming software was used to visualize and shape new shared space and weekend building parties were organized to enable the residents participate in paving, painting spaces, tree planting and transforming children’s play areas | 40 partners including Placemakers Netherlands, the International New Town Institute and design agency KUWA | Reduced crime, improved sanitation, increased children play areas | Challenges include lack of clear financial management and diligence, and power struggles  Planning for unexpected delays would greatly improve initiatives |
| 1. [Jigna Open Space](https://worldlandscapearchitect.com/a-tale-of-two-cities-public-space-development-in-nigeria/?v=518f4a738816) | Abuja, Nigeria  2021 | Open space rejuvenation that included forest restoration, park restoration, protecting riverine corridors and maintaining cultural and archeological assets. | Not reported | It resulted in creation of a nature center and modest sports fields | Not reported |
| 1. [National Non-motorized transport](https://unhabitat.org/ethiopia-plans-safer-streets-for-pedestrians-and-cyclists-during-and-after-the-pandemic) | Addis Ababa, Ethiopia 2020 | Planned improvement of public transport system to promote safety walking and cycling. | UN Environment, UN-Habitat, the Institute for Transportation and Development Policy (ITDP) and the Government of Ethiopia | Not reported | Not reported |
| 1. [Public-space-for-children](https://unhabitat.org/public-space-for-children-inaugurated-in-mozambique) | Maputo and Quelimane, Mozambique 2020 | Assessment, redesigning, construction, and decoration of public places and training activities for children and youth to improve their understanding and experience in the public spaces | UN-Habitat and Maputo and Queliman Municipal Councils | Improvement of the quality of public spaces for children | Not reported |
| 1. [Minecraft Initiative](https://news.un.org/en/story/2020/01/1056432) | Kalobei integrated settlement, Kenya, 2017  (informal settlement) | Workshops were organized in Kalobeyei where host and refugee participants used Minecraft to share ideas and make plans to improve the settlement. Their plans included the provision of shade structures, solar lights, trees and a boda boda station for the settlement. | UN-Habitat, Mojang and Microsoft | Since the workshop over 500 trees have been planted, solar lights have been installed in the community’s public spaces, and the area’s micro-climate has thereby been improved | Not reported |
| 1. [Cycling tourism initiative](https://www.un.org/africarenewal/magazine/november-december-2020/rwanda-invests-cycling-helps-boost-clean-air-and-jobs) | Musanze, Rwanda | Waivers on importation of bicycles and to encourage cycling. Additionally, the city governments have implemented quality footpaths on both sides of many newly constructed urban streets | Rwanda Cycling Federation, Global Green Growth Institute | Increased innovation and entrepreneurship in micro-mobility  Increasing cycling activities | Not reported |
| 1. [Walking and cycling around the world](https://www.unep.org/explore-topics/transport/what-we-do/share-road/walking-and-cycling-around-world) | Burundi 2016 | A National Capacity Building Project and non-motorized transport (NMT) Development project was launched to integrate non-motorized transport and individual motorized transport to improve the efficiency of urban mobility, and to encourage a change in attitude toward NMT and public transport. | UNEP, National Government | There is anticipated improvement in efficiency of urban mobility, and attitude change toward NMT and public transport | A rapid population increase in Burundian cities over the years with vehicle ownership has resulted in considerable increase at the expense of non-motorized transport. The rapid growth has resulted increase in number of motor vehicles and private cars which are associated with road traffic |
| 1. [Share The Road Activities](https://www.unep.org/explore-topics/transport/what-we-do/share-road/walking-and-cycling-around-world) | Addis Ababa, Ethiopia 2020 | Promotion of walking and cycling as a key mode of transport in cities and rural centers | UNEP, UN Habitat, and ITDP, National Government, World Resources Institute (WRI) | Anticipated access to safe, efficient, and accessible walking and cycling networks to improve mobility for all residents, | Not reported |
| 1. [Share The Road Activities](https://www.unep.org/explore-topics/transport/what-we-do/share-road/walking-and-cycling-around-world) | Ghana | Prioritizing of NMT and implementation of share the Road programme. Infrastructure improvements to more effective street management and improved public transport services have been incorporated into the strategy. | Not reported | Not reported | Not reported |
| 1. [Rebuilding The Waterfront](https://www.blockbyblock.org/projects/manakara) | Manakara, Madagascar 2016 | Residents participated in Block-by-Block workshops with an aim of creating a “Parkway Manakarois” along the public beach, developing the waterfront into a recreational space that can be enjoyed by everyone. Architectural designs based on the ideas generated in the workshop were developed into final plans, and the park—including a garden, play equipment, and beach volleyball court were developed | UN-Habitat, Urban Commune of Manakara | The park has resulted in improved community socialization, cohesion | Not reported |
| 1. [Rebuilding Safe Parks](https://www.blockbyblock.org/projects/johannesburg) | Johannesburg, South Africa 2015 | Residents participated in Block-by-Block workshops and used Minecraft to propose upgrading of benches and street lighting, toilets, playgrounds, and an open-air gym. Implementation of the project is ongoing. | UN-Habitat, City of Johannesburg, Ericsson, Johannesburg Development Agency, Wits University | Not reported | Not reported |
| 1. [Designing play spaces](https://thecityateyelevel.com/stories/block-by-block-in-accra-designing-play-spaces-with-urban-youth/) | Accra, Ghana 2017 | Minecraft tool was used to generate ideas for making pockets within the market safer and more playful for the children who accompany their market-vendor-mothers to work. Construction of the spaces is ongoing | UN-Habitat | Not reported | Not reported |
| 1. [Building A Child-Friendly Marketplace In Accra](https://www.blockbyblock.org/projects/accra) | Accra, Ghana 2018 | UN-Habitat established a project to create kid-friendly micro-spaces within Malata Market. A workshop was organized to teach participants to use Minecraft to redesign public spaces. Based on participant’s suggestions, numerous micro-interventions within the market were created. Tires, wooden pallet and other materials were repurposed to create playing space for children | UN-Habitat, HealthBridge, Mmofra Foundation, Tech Needs Girls | Not reported | Not reported |
| 1. [Child play spaces in Malata and Nima markets](https://healthbridge.ca/dist/library/Accra_compressed.pdf) | Accra, Ghana 2018 | Minecraft workshop with 23 community members participating. In total 150 structures were added with installation of play-enabled surfaces (writing surfaces, climbing units, interactive local games) and loose parts to the two markets. | Soronko Solutions, Foundation  for Contemporary Art, Spread-Out Initiative, Mmofra Foundation | The project achieved its goal in creating safe play  spaces for the children of market vendors and  users of limited means | Not reported |
| 1. [Safer streets for pedestrians and cyclists](https://unhabitat.org/ethiopia-plans-safer-streets-for-pedestrians-and-cyclists-during-and-after-the-pandemic) | Addis Ababa, Ethiopia 2020 | The government promoted and invested in walking and cycling through upgrading and implementing footpaths and bicycle lanes through support to harmonize existing street design guidelines and policy processes. It also included contributed to car free days by mobilizing stakeholders and promote street level activities, as well as technical review assistance of designs of ongoing infrastructure projects | Institute for Transportation and Development Policy (ITDP), UN Environment and UN-Habitat | Not reported | Not reported |
| 1. [Connecting People, Cities and the Ocean](https://unhabitat.org/un-habitat-and-unep-jointly-launch-a-new-project-%E2%80%9Cconnecting-people-cities-and-the-ocean-innovative) | Coast region, Kenya 2022 | Not applicable | UNEP, UN-Habitat | Not reported | Not reported |
| 1. [Bicycle signage](https://twitter.com/ludi_org/status/1328310902320492546) | Lagos, Nigeria 2020 | Painting of bicycle signage and installation of bicycle racks | Lagos Urban development Initiative | Not reported | Not reported |
| 1. [Building Space To Gather And Garden](https://www.blockbyblock.org/projects/niamey) | Niamey, Niger 2020 | UN-Habitat partnered with the community and other stakeholders to rehabilitate unusable park lands. Block by block approach using Minecraft was used to visualize the possibilities. A playground with swings, a seesaw, and play space for local children were developed | UN-Habitat, HealthBridge, Peaceful Roads | Not reported | Not reported |
| 1. [Paving way for cyclist](https://www.itdp.org/2018/11/14/paving-way-for-cyclists-in-cairo/) | Cairo, Egypt 2018 | Planned development of 700 bicycle lanes. Workshop carried out to plan the design of the bicycle sharing system | Cairo governorate, UN-Habitat, Drosos Foundation, and ITDP Africa | Not reported | Not reported |
| 1. [Transport matters](https://www.itdp.org/2021/03/17/kisumu-puts-pedestrians-first/) | Kisumu, Kenya 2021 | Kisumu launched Kisumu Sustainable Mobility Plan. This led to development of 1.5 Km of walkways, drainage improvements, street lighting, and installation of utility ducts. | ITDP and UN-Habitat. | Improve safety for pedestrians and cyclists | Full implementation of Kisumu Sustainable Mobility plan will not be possible without proper funding. However, Commitment from the County government will make the improvements possible |
| 1. [Ambitious walking and Cycling](https://www.itdp.org/2019/04/11/addis-ababa-walking-and-cycling-strategy/) | Addis Ababa, Ethiopia 2019 | Planned improvement of public transport system to promote safety walking and cycling that led to construction of about 28km of walkways | Addis Ababa Road and Transport Bureau (AARTB), and ITDP | Improved walking and cycling infrastructure will support pedestrian movement and quality of life | Not reported |
| 1. [Linear Park Project](https://ludi.org.ng/linear-park-project/) | Lagos, Nigeria 2018 | Planned development of 4km off-road bicycle trail and attendant infrastructure, ecological, monumental and recreational parks, bridges and *agritainment* Centre | Lagos State MDAs, Heinrich Boëll Stiftung and the Lagos Urban Development Initiative | Not reported | Not reported |
| 1. [Car free days](https://www.codatu.org/actualites/douala-cameroon-great-success-for-the-car-free-day/) | Douala, Cameroon 2018 | The first car-free day called “the Street for All” was organized by the Community of Douala It included bicycle parade, walks, presentations on road safety, playgrounds | Douala Urban Community, MobiliseYourCity, | The project assisted municipality in the elaboration of its Sustainable Urban Mobility Plan (SUMP) | Not reported |
| 1. [Inner-City safe public space project](https://www.urbanet.info/towards-pan-african-spaces-of-public/) | Johannesburg, South Africa | A pilot project that aimed at transform inner-city parks into safe and inclusive community spaces was implemented. | UN-Habitat Global Public Space Programme, the GIZ Inclusive Violence and Crime Prevention (VCP) Programme, and the local organization Sticky Situations | Not reported | Not reported |
| 1. [Open streets](http://openstreets.org.za/about-open-streets-cape-town) | Cape Town, South Africa | About 12 Open Streets Days in five parts of Cape Town have been held to promote car free days and social activities | City of Cape town, Transformative Urban Mobility Initiative, The World Wide Fund for Nature, DGMT, | Not reported | Not reported |
| 1. [Public Space Programme](https://unhabitat.org/new-model-street-launched-in-dandora-kenya) | Dandora Nairobi, Kenya  (informal settlement) | The project involved creation of a Model Street in Dandora Phase II, including paving and street lighting | UN-Habitat, Dandora Transformation League | -improved security  - Due to paving of the street block, children have now embraced skating as a sport, and some are pursuing it career | Not reported |
| 1. [Share The Road Activities](https://www.unep.org/explore-topics/transport/what-we-do/share-road/walking-and-cycling-around-world) | Addis Ababa, Ethiopia 2020 | Promotion of walking and cycling as a key mode of transport in cities and rural centers | UNEP, UN Habitat, and ITDP, National Government, World Resources Institute (WRI) | Anticipated access to safe, efficient, and accessible walking and cycling networks to improve mobility for all residents, | Not reported |
| 1. [Share The Road Activities](https://www.unep.org/explore-topics/transport/what-we-do/share-road/walking-and-cycling-around-world) | Accra, Ghana | Prioritizing of Non-Motorized Transport (NMT) and implementation of share the Road programme.  Infrastructure improvements to more effective street management and improved public transport services have been incorporated in to the strategy. | Not reported | Not reported | Not reported |
| 1. [Share the road](https://www.unep.org/explore-topics/transport/what-we-do/share-road/walking-and-cycling-around-world) | Nairobi, Kenya 2012 | Development of transit and pedestrian-friendly infrastructure | Nairobi County Government, UNEP, FIA | Not reported | Not reported |
| 1. [Share the road](https://www.unep.org/explore-topics/transport/what-we-do/share-road/walking-and-cycling-around-world) | Lagos, Nigeria 2013 | Launching of The Lagos Metropolitan Transport Authority (LAMATA) 2013 Safety plan that recognizes non-motorized transport for improved walking and cycling. Improved station design and passenger access elements is being implemented | Not reported | Not reported | Not reported |
| 1. [Share the road](https://www.unep.org/explore-topics/transport/what-we-do/share-road/rwanda) | Rwanda | Construction of pedestrian foot paths and more focus on walking, cycling, car free days and public transport | Government of Rwanda, | Not reported | Not reported |
| 1. [Share the road](https://www.unep.org/explore-topics/transport/what-we-do/share-road/uganda) | Kampala, Uganda 2012 | Development of non-motorized transport to increase the recognition of walking and cycling in transport planning and design, revolving around provision of safe infrastructure for pedestrians and cyclists. As a pilot, a 2-kilometer stretch of road that cuts through the city was remodeled. The ‘pedestrian shopping streets’ that involves having the Namirembe road and old Luwum Street redesigned into a car-less space with walking paths and wider green spaces for recreation. | Kampala Capital City Authority (KCCA) | Not reported | Not reported |
| 1. [Share the road](https://www.unep.org/explore-topics/transport/what-we-do/share-road/zambia) | Lusaka, Zambia | Development of NMT Strategy which has ensured more equitable allocation road space by focusing on walking, cycling, and public transport and prioritizing the needs of cyclist and pedestrians. It has led to development of high-quality footpaths such as construction of 40km of complete pedestrian facilities and 20km cycle tracks per year across ten cities and towns | The Government of Zambia, UNEP, UNDP Zambia | Not reported | Not reported |
| 1. [Walking and Cycling around the World](https://www.unep.org/explore-topics/transport/what-we-do/share-road/cote-divoire) | Abidjan, Côte d’Ivoire 2015 | Development of the Urban Master Plan in Greater Abidjan that raised Concerns about pedestrian safety and urban quality are frequently raised was launched. It proposed development of a network of sidewalks and bicycle paths that provide interior circulation, as well as connections to nearby schools, shops. | Ministry of Construction, Housing, Sanitation and Urban Development (MCLAU), JICA (Japan International Cooperation Agency | Not reported | Not reported |
| 1. [Walking and Cycling around the World](https://www.unep.org/explore-topics/transport/what-we-do/share-road/madagascar) | Madagascar 2015 | Development of a training curriculum for bicycle assembly, maintenance, repairs and management of bicycles for community health workers | Transaid, Malagasy NGO Lalana | It has enabled community health workers to visit more people in a day, travel further, and attend to emergencies quicker |  |
| 1. [Walking and Cycling around the World](https://www.unep.org/explore-topics/transport/what-we-do/share-road/malawi) | Malawi 2014 | Revision of The National Transport Policy that advocates for specific consideration of NMT users to ensure that walking and cycling are promoted as healthy, sustainable, economical and non-polluting means of transport | Not reported | Not reported | Not reported |
| 1. [Walking and Cycling around the World](https://www.unep.org/explore-topics/transport/what-we-do/share-road/mozambique) | Maputo, Mozambique 2015 | A Maputo Bicycling Safety and Mobility Study was conducted by the two partners which included a pre-feasibility study for a bicycle lane network in Maputo, and the potential for incorporating bicycle facilities within the planned BRT corridor | Mozambikes, Alta Planning and Design | Not reported | Not reported |
| 1. [Walking and Cycling around the World](https://www.unep.org/explore-topics/transport/what-we-do/share-road/namibia) | Windhoek, Namibia | Development of a Sustainable Urban Transport Master Plan (SUTMP) for the capital with an aim of providing efficient, affordable, equitable, safe and convenient public and NMT for residents of the city and its surroundings. NMT strategy was developed to create safe and pedestrian friendly transport system, infrastructure for pedestrian and cyclist, improve pedestrian network and encourage cycling | Ministry of Works and Transport | Not reported | Not reported |
| 1. [Walking and Cycling around the World](https://www.unep.org/explore-topics/transport/what-we-do/share-road/south-africa) | South Africa 2014 | Qhubeka organization supplied bicycles to people who need them in return for work done to improve the community, environment or academic result. Moreover, in 2014 South Africa developed, revised and updated of the existing Pedestrian and Bicycle Facility Guidelines (2003) and developed a new NMT Facility Guidelines that aim to improve the lives of all South Africans | Qhubeka Organization | Not reported | Not reported |
| 1. [Walking and Cycling around the World](https://www.unep.org/explore-topics/transport/what-we-do/share-road/tanzania) | Dar es Salaam, Tanzania | In 2011 a new national transport policy was drawn up and it emphasizes rural NMT and IMT modes as well as urban pedestrians although makes no mention of bicycle transport. Over the recent years, Dares Salaam has launched a series of transformative improvements to transit, cycling and walking | International Development (DFID), World Bank | DART project led to development of cycle paths, sidewalks, and improved pedestrian safety with well-designed, at-grade pedestrian crossings also complying with universal accessibility principles | Not reported |
| 1. [Jeevanjee Gardens and Silanga Community](https://unhabitat.org/sites/default/files/download-manager-files/Public%20Space%20Programme%20Annual%20Report%202017.pdf)   [Complex,, Page 50](https://unhabitat.org/sites/default/files/download-manager-files/Public%20Space%20Programme%20Annual%20Report%202017.pdf) | Nairobi, Kenya 2012 | Participatory design workshop was carried out to upgrade Jeevanje Gardens and Silanga site. New pathways, street furniture, waste bins  and entrances were put up in Jeevanje. | UN-Habitat and Nairobi City Council | -The County has embraced public space as an important political agenda | For Silanga site, it was disagreement on joint management between a local partner and the community |
| 1. [Model street, Dandora, Page 51](https://unhabitat.org/sites/default/files/download-manager-files/Public%20Space%20Programme%20Annual%20Report%202017.pdf) | Dandora, Nairobi Kenya 2015  (informal settlement) | Partners teamed up to revitalize Dandora public spaces. First 33 court yards were upgraded by engaging youth groups through competition. | UN-Habitat, KUWA and Dandora Transformation League | The project has resulted in upgrading of street, clearing of ditches and trees planted | Not reported |
| 1. [Industrial Area revitalization, Page 52](https://unhabitat.org/sites/default/files/download-manager-files/Public%20Space%20Programme%20Annual%20Report%202017.pdf) | Industrial area, Nairobi | Participatory urban design and planning for public space development. | UN-Habitat, GoDown Arts Centre and White Architects | Not reported | Not reported |
| 1. [Pedestrianisation project, Page 53](https://unhabitat.org/sites/default/files/download-manager-files/Public%20Space%20Programme%20Annual%20Report%202017.pdf) | Ruiru, Nairobi 2016 | Design workshops were carried out transform public space. Pedestrian space was reclaimed by redesigning street, intersections and parking spaces. Spaces were designated for street vendors and roads paved | UN-Habitat, Ruiru County Government | Not reported | Not reported |
| 1. [Preparanda square, Page 54](https://unhabitat.org/sites/default/files/download-manager-files/Public%20Space%20Programme%20Annual%20Report%202017.pdf) | Lokoja, Nigeria 2014 | A participatory planning and design workshop using Minecraft. This was followed by up-grading and revitalization of Preparanda Square and involved improving access to public space and quality urban life, securing the recreational aspect of the facilities and providing a space for youth to meet and engage whilst also creating job opportunities through public space rehabilitation. | State Government, UN-Habitat | Not reported | Not reported |
| 1. [End Street North Park, Page 55](https://unhabitat.org/sites/default/files/download-manager-files/Public%20Space%20Programme%20Annual%20Report%202017.pdf) | Johannesburg, South Africa 2015 | A participatory design workshop using Minecraft was held which led to emphasis interventions such as upgrading of street furniture, toilets, playgrounds and sports equipment | Johannesburg City Parks and Zoo Department, UN-Habitat | Scarce resource to facilitate the implementation | collaborative park design or upgrade process will create a sense of ownership  amongst park users, reducing vandalism, theft and other illicit activities in the park. |
| 1. [Parkway, Mankarois, Page 56](https://unhabitat.org/sites/default/files/download-manager-files/Public%20Space%20Programme%20Annual%20Report%202017.pdf) | Manakara, Madagascar 2016 | Block by Block community participation workshop were held to create a public space “Manakarois” along the public beach, developing the waterfront into a recreational public space | UN-Habitat, Urban Commune of Manakara | Not reported | Not reported |
| 1. [Public space planning, Page 56](https://unhabitat.org/sites/default/files/download-manager-files/Public%20Space%20Programme%20Annual%20Report%202017.pdf) | Bamenda, Cameroon 2016 | A two-day training workshop in which residents, students and local government officials learnt how to use UN-Habitat’s public space assessment tool was held. Public Space and Marketplace Programme were launched after participants mapped out 86 public spaces and markets | UN-Habitat and Bamenda City Council | Not reported | Not reported |
