# Supplementary material for: Urban public space initiatives and health in Africa: A mixed-methods systematic review
Source: PLOS Glob Public Health. 2024 Oct 15;4(10):e0003709. doi: 10.1371/journal.pgph.0003709 (PMC11478912; doi:10.1371/journal.pgph.0003709)
Supplement: S1 Text — (DOCX) [file pgph.0003709.s011.docx]

**A systematic review of opportunities for health promotion via public space initiatives in African cities**

**Data extraction template**

**November 2020**

| **Field** | **Description** |
| --- | --- |
| Urban or rural | Was this initiative carried out in an urban or rural setting? If rural, stop here and recommend for exclusion |
| Explicit opportunities to promote health | Describe any component of the initiative that is currently being used to promote physical and/or mental health e.g., a community festival could promote physical activity, and social cohesion, a street food festival could have the potential to promote healthy eating. |
| Implicit opportunities to promote health | Describe opportunities implicit in the initiative that could be used to promote health in the future (even if not mentioned by authors). e.g. urban infrastructure projects in informal settlements may be used to promote opportunities for safe walking |
| Explicit health risks | Please list any health-related risks that were associated with the initiative and explicitly mentioned in the paper e.g., exposure to pollution |
| Health risks considered by the reviewer | Please list any health risks you think could be associated with the initiative even if they were not mentioned in the paper e.g. exercising under bridges may involve risk for exposure to violence and traffic injury |
| Sponsorship source | Enter the institution(s) that sponsored the paper (if any) i.e., who funds the authors and the research e.g., National Institutes of Health Research |

| Date of data collection | Enter the start and end year of data collection  *Examples*  2014  2014-2015 (one discrete period of data collection)  2014, 2017 (multiple periods of data collection e.g., cohort)  2015-2015, 2017-2018 (multiple periods of data collection, each spanning multiple years) |
| --- | --- |
| Date(s) during which the initiative took place (if provided) | *Examples*  2014  2014-2015 (one discrete period of data collection)  2014, 2017 (multiple periods of data collection e.g., cohort)  2015-2015, 2017-2018 (multiple periods of data collection, each spanning multiple years) |
| Date of publication | Enter the year that the study was published  *Example*  2014 |
| **Author contact details** |  |
| Name of the author | Enter the surname and initials of the first author  *Example* Oyeyemi, A.L. |
| Authors’ institutional affiliation | Enter the institutional affiliation of each of the authors, with the country of affiliation in brackets  If more than one, list separated by a comma  *Example*  University of Maiduguri (Nigeria), University of Cape Coast (Ghana) |
| Email address of the corresponding author | Enter the email address of the first author or ‘email not provided’ where this is not available  *Example*  andreowerneck@gmail.com |
| Country of affiliation of the first author | Enter the country or countries of the institution(s) where the first author has an affiliation, separating them with a comma where multiple  *Example*   - Kenya, South Africa |

| Country of affiliation of the last author | Enter the country where the last author’s institution or affiliation is located  If more than one, list the country of the first affiliation  If the study has only one author, enter ‘no last author’  *Example*  United States of America |
| --- | --- |
| Data source | What categories of people were involved in gathering the knowledge generated or information described? Select all of the following that apply   - Academic organizations e.g. the University of Lagos, University of Nsukka - Research Institute/Centre e.g. the African Population and Health Research Center, Alex Ekwueme center for interdisciplinary research - Government e.g. Government of Ekiti State, community health workers in Awka South local government area - Private sector e.g. Julius Berger, Dangote - International non-profit organisation e.g. the Gates Foundation, Tony Elumelu Foundation, - Regional non-profit organization e.g. the African Union, the Economic Community of West African States - Local non-profit organization or civil society group e.g. Alabiamo Foundation, Mentally Aware Nigeria - Community groups or their representatives e.g Obuwanne age grade, Amawbia; residents of Jakande Estate - Other |
| Specific name of the data source | Enter the specific source of the data e.g. Anambra State Ministry of Education |
| **About the initiative** |  |
| Country of implementation or planned implementation | Enter the name of the country where the initiative was carried out, separating multiple countries with a comma e.g. Ghana, Nigeria |
| Where does this initiative take place? | Select all that apply  - Car park  - Bridge locations  - On the road  - Road junctions  - Roadsides  - Market  - Squares  - In sport and recreation clubs e.g. deux zeros football clubs, Cycology, Truppr, football leagues - Waterways  - Roundabouts  - Beach locations (pop beach club)  - Designated fields e.g. FHA fields  - In groups organized by trainers and coaches e.g. Gbagada General Hospital, Magodo Football Club, martials arts, Sunday morning football club  - In residences or community associations e.g. in gated closes, or on the street  - Other (please specify) |

| How would you characterize the land use dimensions of this place? | Here are examples you may use. Select as many as apply  - It is a residential area  - It is an industrial area  - It is an educational area  - It is a dumpsite or waste area  - It is a transitory area e.g. roads, bridges, etc.  - Other (please specify) |
| --- | --- |
| What is the nature of the space? | - A fixed space designated for the initiative e.g. a swimming pool, car park  - An appropriated space e.g. in the middle of the road |
| How did people participate in the initiative? | - As part of a group  - Individually  - Both (e.g. open streets events can be attended individually and in groups) |
| What is the nature of initiatives in this space? | - They happen in an adhoc fashion e.g. people play football there at any time of the day  - They happen in an scheduled fashion e.g. a coach lead a group to run there at a fixed time of the morning, an open street event scheduled on the road at set times of the week/month |
| What explicitly mentioned principles guided the decision to run the initiative? | Examples include healthy cities (including physical and mental wellbeing), social justice, community cohesion, health equity, physical activity, public participation, right to the city etc. |

| Partner(s) involved in  designing the initiative (if  provided) | Select all of the following that apply  - Academic organizations e.g. the University of Lagos, University of Nsukka  - Research Institute/Centre e.g. the African Population and Health Research Center, Alex Ekwueme center for interdisciplinary research  - Government e.g. Government of Ekiti State, community health workers in Awka South local government area ● Private sector e.g. Julius Berger, Dangote  - International non-profit organization e.g. the Gates Foundation, Tony Elumelu Foundation  - Regional non-profit organization e.g. the African Union, the Economic Community of West African States  - Local non-profit organization or civil society group e.g. Cycology, Truppr  - Community groups or their representatives e.g. Obunwanne age grade, Amawbia; residents of Jakande Estate, communities living under the bridge  - Individuals e.g. gym trainers, sports coaches  - Other |
| --- | --- |
| Name of the partner(s) involved in designing the initiative | Write down the name of partner(s) involved in designing the project. Separate multiple partners with a comma e.g. Johnson and Johnson, Dangote Foundation |
| Sector(s) involved in designing the initiative | This refers to the sectors that the partners you just mentioned above represent. Separate multiple entries with a comma  e.g. Ministry of Transport is from the transport sector, Johnson and Johnson from healthcare, the local government chairman from governance, and the World Bank from finance etc. |
| Planetary health considerations in running the initiative | What planetary health considerations did the authors mentions were given to the design and implementation of the initiative? Planetary health concerns the health impacts of disruptions in the earth’s natural systems e.g. water bodies, green infrastructure (e.g. parks), biodiversity, greenhouse gas emissions and climate change, clean oceans etc. e.g. the cycling group was set up to provide clean transportation options |
| Partner(s) involved in funding the initiative | This includes not only the sponsor of the paper which you noted in the beginning, but also the funder(s) of every cycle of the initiative - the design, implementation and evaluation.  Select all of the following that apply  - Academic organizations e.g. the University of Lagos, University of Nsukka  - Research Institute/Centre e.g. the African Population and Health Research Center, Alex Ekwueme center for interdisciplinary research  - Government e.g. Government of Ekiti State, community health workers in Awka South local government area ● Private sector e.g. Julius Berger, Dangote  - International non-profit organization e.g. the Gates Foundation, Tony Elumelu Foundation  - Regional non-profit organization e.g. the African Union, the Economic Community of West African States ● Local non-profit organization or civil society group e.g. Cycology  - Community groups or their representatives e.g. Obunwanne age grade, Amawbia; residents of Jakande Estate, communities living under the bridge  - Individuals e.g., gym trainers, sports coaches  -Other |

| Name of the partner(s)  involved in funding the initiative | This includes not only the authors of the paper which you noted in the beginning, but also the partners(s) who funded every cycle of the initiative. Write down the name of partner(s) involved in funding the initiative. Separate multiple partners with a comma e.g., Johnson and Johnson, Dangote Foundation |
| --- | --- |
| Sector(s) involved in funding the initiative | This refers to the sectors that the multisectoral partners you just mentioned above represent, Separate multiple entries with a comma e.g., transport, health, governance |
| Planetary health  considerations in funding the initiative | What planetary health considerations were given to funding the initiative? Planetary health concerns the health impacts of disruptions in the earth’s natural systems.e.g., the government awarded the contract for the project to the bidder with the best environmental footprint |
| Partner(s) responsible for implementing the initiative | This includes not only the authors of the paper which you noted in the beginning, but also the partners(s) who implemented every cycle of the initiative. Select all of the following that apply  - Academic organizations e.g. the University of Lagos, University of Nsukka  - Research Institute/Centre e.g. the African Population and Health Research Center, Alex Ekwueme center for interdisciplinary research  - Government e.g. Government of Ekiti State, community health workers in Awka South local government area ● Private sector e.g. Julius Berger, Dangote  - International non-profit organization e.g. the Gates Foundation, Tony Elumelu Foundation  - Regional non-profit organization e.g. the African Union, the Economic Community of West African States ● Local non-profit organization or civil society group e.g. Cycology  - Community groups or their representatives e.g. Obunwanne age grade, Amawbia; residents of Jakande Estate, communities living under the bridge  - Individuals e.g. gym trainers, sports coaches  - Other |
| Name of the partner(s)  involved in implementing the initiative | Write down the name of partner(s) involved in implementing the initiative. Separate multiple partners with a comma e.g. Johnson and Johnson, Dangote Foundation |

| Sector(s) involved in  implementing the initiative | This refers to the sectors that the multisectoral partners you just mentioned above represent, Separate multiple entries with a comma e.g., transport, health, governance |
| --- | --- |
| Planetary health  considerations in implementation the initiative | What planetary health considerations were given to the implementation of the initiative? Planetary health concerns the health impacts of disruptions in the earth’s natural systems. e.g., project resources were sourced locally to minimize the environmental imprint from air and road transportation |
| Partner(s) involved in  evaluating the initiative | This includes not only the authors of the paper which you noted in the beginning, but also the partners(s) who evaluated every cycle of the initiative. Select all of the following that apply  - Academic organizations e.g. the University of Lagos, University of Nsukka  - Research Institute/Centre e.g. the African Population and Health Research Center, Alex Ekwueme center for interdisciplinary research  - Government e.g. Government of Ekiti State, community health workers in Awka South local government area - Private sector e.g. Julius Berger, Dangote  - International non-profit organization e.g. the Gates Foundation, Tony Elumelu Foundation,  - Regional non-profit organization e.g. the African Union, the Economic Community of West African States  - Local non-profit organization or civil society group e.g. Alabiamo Foundation, Mentally Aware Nigeria  - Community groups or their representatives e.g. Obunwanne age grade, Amawbia; residents of Jakande Estate  - Other |
| Name of the partner(s)  involved in evaluating the initiative | Write down the name of partner(s) involved in evaluating the project. Separate multiple partners with a comma e.g Johnson and Johnson, Dangote Foundation |
| Sector(s) involved in  evaluating the initiative | This refers to the sectors that the multisectoral partners you just mentioned above represent, Separate multiple entries with a comma e.g., transport, health, governance |
| Planetary health  considerations in evaluating the initiative | What planetary health considerations were given to the evaluation of the project? Planetary health concerns the health impacts of disruptions in the earth’s natural systems. e.g. a health impact assessment was included in the economic evaluation of the park |
| Partner(s) involved in  advocating for the initiative | This includes not only the authors of the paper which you noted in the beginning, but also the partners(s) who advocated for every cycle of the initiative. Select all of the following that apply  - Academic organizations e.g. the University of Lagos, University of Nsukka |

|  | - Research Institute/Centre e.g. the African Population and Health Research Center, Alex Ekwueme center for interdisciplinary research  - Government e.g. Government of Ekiti State, community health workers in Awka South local government area  - Private sector e.g. Julius Berger, Dangote  - International non-profit organization e.g. the Gates Foundation, Tony Elumelu Foundation,  - Regional non-profit organization e.g. the African Union, the Economic Community of West African States - Local non-profit organization or civil society group e.g. Cycology,  - Community groups or their representatives e.g. Obunwanne age grade, Amawbia; residents of Jakande Estate, communities living under the bridge  - Individuals e.g. gym trainers, sports coaches  - Other |
| --- | --- |
| Name of the partner(s)  involved in advocating for the initiative | Write down the name of partner(s) involved in advocating the project. Separate multiple partners with a comma e.g.Johnson and Johnson, Dangote Foundation |
| Sector(s) involved in advocating for the initiative | This refers to the sectors that the multisectoral partners you just mentioned above represent, Separate multiple entries with a comma e.g. transport, health, governance |
| Planetary health  considerations in advocacy for the initiative | What planetary health considerations were given to advocacy for the project? Planetary health concerns the health impacts of disruptions in the earth’s natural systems. e.g. the community requested the project because of the high rates of diabetes in their town |
| Country where the initiative was implemented | Separate multiple entries with a comma e.g. Kenya, Nigeria |
| State or province where the initiative was implemented | Separate multiple entries with a comma  e.g. Lagos, Bungoma county |
| Town or locality | Separate multiple entries with a comma  e.g. Agege, Webuye municipality |
| Setting(s) of the initiative | This refers to where the initiative was implemented. Separate multiple entries with a comma e.g. parks, the home, the school, church, mosque, marketplace |
| Informality or formality of the setting | Note the nature of the setting(s). Select both if applicable   - Formal - Informal - Not mentioned |

| Outcome of the initiative | What was the desired or measured outcome of the initiative?  Choose as many as apply, specifying the particular outcome in brackets   - Health behaviours - Health outcomes - Developing collaborations - Improving the built environment - Improving the natural environment - Providing social infrastructure to improve participation and empowerment of community members e.g. creation of a community group to support the mental health of teenagers - Access to services and physical infrastructure e.g. providing free library cards for mothers - Other (specify):............ |
| --- | --- |
| Specify the findings on the outcome(s) of the initiative (if any) | Separate multiple with a comma e.g. improved physical activity, reduced diabetes, improved social cohesion, improved mental health |
| What population was the initiative targeted at? | Separate multiple with a comma e.g. pregnant women in Sogunro community, children |
| What population was the initiative targeted at? | Separate multiple with a comma e.g. pregnant women in Sogunro community, children |
| Outcome measurement | Was the outcome measured?   - Yes - No |
| Outcome measurement method | If your answer above was yes, enter used to assess the outcomes:   - Subjective (Usually this means the participant in the initiative has self-reported) - Objective (Observed by the researcher. For example, counting people in the group) - Both subjective and objective |
| Outcome method specification | Specify how the outcome was measured e.g. through researcher led observation of weekly physical activity at the new park |
| Analysis method - quantitative | Enter a small amount of free text summarising the analysis methods. For quantitative studies it is important to distinguish between basic statistical methods for describing quantities of variables (e.g. modes, means, standard deviations), assessing relationships (e.g. correlations) and those that account for potential confounding factors (e.g. multivariable regression modelling). For quantitative or mixed method studies, complete this field. Otherwise (i.e. for qualitative studies) enter ‘not applicable’ |
| Analysis method - qualitative | Enter a small amount of free text summarising the analysis methods, e.g. thematic analysis.  For qualitative or mixed method studies, complete this field. Otherwise (i.e. for quantitative studies) enter ‘not applicable’ |

| Long term considerations for sustaining the initiative | While the previous entry details the short-term implementation of the project, this entry concerns the considerations for the long-term sustainability mentioned in the article. For example, these could be policies, procedures and activities put in place to ensure the project could go on long term, or challenges faced that could limit the project’s continuity, etc |
| --- | --- |
| Lessons learned | Note any lessons learned, limitations or challenges noted by the authors throughout the cycle of the initiative - from its design, implementation, evaluation and advocacy for the project, that you think could inform future replication even though they do not fall under the barriers and facilitators listed above  Here are some examples:   - Participants in regular exercise sessions at the Federal Housing Authority field reported improved mental health, reduced obesity and benefited from the opportunity for social interaction. However, they also noted potential challenges such as harassment by “area boys” and a planned gate fee for using the field. Future activities such as these will need to overcome formal and informal governance challenges that arise with increasing mass participation. - religious groups contributed in advocating for the initiative at the local government level - funding limitations made it impossible to get equipment to continue the initiative - a change in government led to a reappropriation of the space, thus making the initiative impossible - evidence was useful in convincing policy makers to allow for community gardening on the curbs |
| **Study methods** |  |
| Research type | Enter the research type used to assess the initiative:  Use ONE of the following categories:  - Quantitative (measures of values or counts expressed as numbers e.g. the data generated from surveys, questionnaires, measurements)  - Qualitative (description of phenomena often expressed as text e.g. interviews, focus groups) - Mixed method (Both quantitative and qualitative methods used) |
| Study design - quantitative | Enter the study design.  Quantitative or mixed method study - use ONE of the following categories: |

|  | - Randomised controlled trial - Cohort study - Case-control study - Cross sectional study - Case report / Case study - Other – please, specify   For quantitative or mixed method studies, complete this field. Otherwise (i.e. for qualitative studies) enter ‘not applicable’ |
| --- | --- |
| Study design - qualitative | Enter the study design.  Qualitative or mixed method study – use ONE of the following categories:  - Grounded theory  - Case study  - Historical / Narratives  - Participatory research / Action research  - Phenomenology  - Ethnography / Observation  - Other – please, specify  Ethnography: Immersion of the researcher the participants’ environment, typically through observation (note, do not confuse this design with observational quantitative designs like traffic counting). For qualitative or mixed method studies, complete this field. Otherwise (i.e. for quantitative studies) enter ‘not applicable’ |
| Study method – quantitative | If the study includes a quantitative element, enter the method type. Enter a small amount of free text summarising the study methods. This can usually be cut and pasted from the abstract.  *Examples*   - Two-day vox pop survey, structured questionnaire at two main trip destinations - STEPs survey - Household survey and interview   For quantitative or mixed method studies, complete this field. Otherwise (i.e. for qualitative studies) enter ‘not applicable’ |
| Study method - qualitative | If the study includes a qualitative element, enter the method type. |

|  | Use the following categories:  If more than one, list separated by a comma   - Structured – interview, survey, questionnaire - Un-structured or Semi structured - interview, survey, questionnaire - In depth- interview / Key informants - Focus groups / Group discussions - Field Notes - Narrative descriptions - Audio tapes - Video tapes - Seasonal calendars - Transect walks - Participatory mapping / Modelling - Other– please, enter a small amount of free text summarising the study methods.   For qualitative or mixed method studies, complete this field. Otherwise (i.e. for quantitative studies) enter ‘not applicable’ |
| --- | --- |
| Inclusion criteria (if relevant) | Enter a small amount of free text summarising the types of participants included in the intervention. This can usually be cut and pasted from the article.  *Example*   - Residents in 34 communities who had weekly training on kickboxing |
| Exclusion criteria | Enter a small amount of free text summarising the types of participants excluded from the intervention. This can usually be cut and pasted from the article. In many cases, there will be no explicit exclusion criteria listed. If this is the case, enter ‘not reported’.  *Example*  Participants with a diagnosis of cancer were excluded from the intervention |

| Group differences | Enter a small amount of free text summarising whether there were any differences between participant groups at baseline. This only applies to studies that used a controlled experimental design (and it is likely that the majority of literature identified will not use this type of design). This can usually be cut and pasted from the article. If this does not apply, enter ‘not applicable’. |
| --- | --- |
| **Additional population data** |  |
| Sample size | Enter a small amount of free text describing the sample size  *Examples*   - 10,128 individuals - 100 households, 1 member of each household - Ouagadougou - 754 households, 3682 individuals, Bamako - 251 households, 1666 individuals |
| Response rate | Enter a small amount of free text describing the response rate, if provided. If reported, this is typically expressed as a percentage. It is likely that many studies will not provide this information. If not reported, enter ‘not reported’  *Example*   - 78% response rate |
| Age | Enter a small amount of free text describing the age of participants. If possible, enter this in the format ‘mean or median (standard deviation)’. Other common reporting formats are a range, or the proportion of participants that fall within a particular age range.  If not reported, enter ‘not reported’  *Examples*   - 45.3 (2.1) years - 15-60 years - 48% of participants were aged 30-44 years - Over 13 years |

| Sex | Enter a small amount of free text describing the sex of participants. If possible, enter this in the form ‘number (percentage)’ If not reported, enter ‘not reported’  *Examples*   - 100 (25%) female; 300 (75%) male - 55% female - Female only |
| --- | --- |
| Direction of relationships | Enter a small amount of free text summarising the direction of the relationships found between exposures and outcomes. Try to keep this as succinct as possible, although it is likely that some studies will report a large number of relationships. Do not worry about reporting the size of the relationship.  *Examples*   - Communities with physical activity groups had less hypertension |
| Main idea | Imagine you would like to summarise the article to a colleague in a few sentences. What would you tell him/her? Try to get the main idea of the article. Limit your answer to 1 paragraph containing 3 to 5 sentences (maximum) |
