# Supplementary material for: Urban public space initiatives and health in Africa: A mixed-methods systematic review
Source: PLOS Glob Public Health. 2024 Oct 15;4(10):e0003709. doi: 10.1371/journal.pgph.0003709 (PMC11478912; doi:10.1371/journal.pgph.0003709)
Supplement: S2 Text — (DOCX) [file pgph.0003709.s012.docx]

**Characteristics of included studies**

| **Author (year)** | **Region** | **Country (setting)** | **Quantitative methods** | **Qualitative methods** | **Focus of the initiatives** | **Sample characteristics** | **Funding** | **Authorship** |
| --- | --- | --- | --- | --- | --- | --- | --- | --- |
| **Qualitative studies** | | | | | | | | |
| Cubizolles, S., 2010. Soccer in a rugby town: restructuring football in Stellenbosch. Soccer & Society, 11(1-2), pp.105-117. | Southern Africa | South Africa (Stellenbosch) | Not Applicable | Semi-structured interviews | Developing collaborations between local soccer clubs and their players and officials | Sample size: n=12 individuals  Area: urban  Sex: Not reported  Age: Not reported | University of Reunion, Island of Reunion, France | First author: France  Last author: N/A |
| Cubizolles, S., 2012. Integrating a popular sport into the patrimony in a South African provincial town: The case of football in Stellenbosch. *African Studies*, *71*(1), pp.108-126. | Southern Africa | South Africa (Stellenbosch) | Not Applicable | Structured interview, survey, questionnaire, and narrative descriptions | Sports promotion, tourism | Sample size: n=26 individuals  Area: urban  Sex: Not reported  Age: Not reported | University of Reunion, Island of Reunion, France | First author: France  Last author: N/A |
| Chaminuka, N. and Makaye, P., 2015. The Resilience of Urban Agriculture in the Face of Adversity from the City Authorities: The Case of mkoba. *Global J. Hum. Soc. Sci.: interdisciplinary*, *15*(3). | Southern Africa | Zimbabwe (Gweru) | Not Applicable | In depth- interviews with key informants | Improved health and nutrition, increased income, employment creation. | Sample size: n=Not reported  Area: urban  Sex: Not reported  Age: Not reported | No funding reported | First author: Zimbabwe  Last author: Zimbabwe |
| Amanda, B., 2007. Masibambane-lets stick together': contentions on the role of urban vegetable gardens in the Cape Flats (Master's thesis, University of Cape Town). | Southern Africa | South Africa (Cape town) | Not Applicable | Un-structured or Semi structured - interviews, survey, questionnaire | Nutrition | Sample size: n=18  Area: urban  Sex: 20% males, 80% female  Age: Older adults | University of Cape Town | First author: South Africa  Last Author: Not applicable |
| Brady, M. and Khan, A.B., 2002. Letting Girls Play: The Mathare Youth Sports Association's Football Program for Girls. | Eastern Africa | Kenya (Nairobi) | Not Applicable | In depth- interview, Key informants, field visits | Health behaviours, Providing social infrastructure | Sample size: n=Not reported  Area: urban informal settlement  Sex: 100% female  Age: adolescents | Gates and Turner Foundations, the Cabot Family Charitable Trust, and the Population Council | First author: United States of America  Last Author: Kenya |
| Webb, N.L., 2000. Food-gardens and nutrition: Three Southern African case studies. Journal of Consumer Sciences, 28. | Southern Africa | South Africa, Zimbabwe | Not applicable | Case studies | Food security and increased nutrition | Not applicable | Not reported | First author:  South Africa  Last author: Not applicable |
| Omondi, S.O., Oluoch‐Kosura, W. and Jirström, M., 2017. The role of urban‐based agriculture on food security: Kenyan case studies. Geographical research, 55(2), pp.231-241. | Eastern Africa | Kenya(Kisumu, Thika) | Not applicable | Structured - interview, survey, questionnaire, field notes, transect walks | Food security | Sample size: n=2009 households  Area: urban  Sex: Not reported  Age: adolescents | The Swedish Research Council (VR) and the Swedish Research Council for Environment, Agricultural Sciences and Spatial Planning(Formas) | First author:  Kenya  Last author: Sweden |
| Kanosvamhira, T.P., 2019. The organisation of urban agriculture in Cape Town, South Africa: A social capital perspective. Development Southern Africa, 36(3), pp.283-294. | Southern Africa | South Africa (Cape Town) | Not applicable | Review | Developing collaborations, mobilization of external resources, sharing of ideas, and improving the natural environment. | Not reported | German Federal Agency for Food and Agriculture (BLE). | First author:  South Africa  Last author: Not applicable |
| Wesselow, M., 2019. “Who Needs Money if You Got Hands, if You Got Plants” Forming Community Resilience in Two Urban Gardening Networks in South Africa. Human Ecology, 47(6), pp.855-864. | Southern Africa | South Africa (Western Cape) | Not applicable | Semi-structured interviews, observations and focus group | Food security | Sample size: 40 members  Area: urban  Sex: not reported  Age: not reported | Federal Ministry of Education and Research (BMBF) | First author: Germany  Last author: South Africa |
| Reuther, S. and Dewar, N., 2006. Competition for the use of public open space in low-income urban areas: the economic potential of urban gardening in Khayelitsha, Cape Town. Development Southern Africa, 23(01), pp.97-122. | Southern Africa | South Africa (Vacant land) | Not applicable | Literature review | Securing household food security | Sample size: Not reported  Area: urban  Sex: not reported  Age: not reported | Not reported | First author:  South Africa  Last author: South Africa |
| Cubizolles, S., 2015. Sport and social cohesion in a provincial town in South Africa: The case of a tourism project for aid and social development through football. International Review for the Sociology of Sport, 50(1), pp.22-44. | Southern Africa | South Africa (Western Cape) | Not applicable | Semi-structured interviews | Improving the built environment, providing social infrastructure | Sample size: n= 18  Area: urban  Sex: males only  Age: footballers aged 22-25 years & club officials aged 32-45 years | No funding reported | First author: N/A  Last author: N/A |
| Koloba, H.A. and Surujlal, J., 2014. Factors and challenges associated with participation in community sport in Eldorado Park, Johannesburg, South Africa. *Mediterranean Journal of Social Sciences*, *5*(20), p.30. | Southern Africa | South Africa (Gauteng) | Not applicable | Semi- structured interviews, audio tapes, thematic analysis | Providing social infrastructure and the built environment | Sample size: n=15  Area: urban  Sex: not reported  Age: not reported | North-West University, Vanderbijlpark | First author: South Africa  Last author: South Africa |
| Tembo, R. and Louw, J., 2013. Conceptualising and implementing two community gardening projects on the Cape Flats, Cape Town. Development Southern Africa, 30(2), pp.224-237. | Southern Africa | South Africa (Western Capee) | Not applicable | Semi- structured interviews, focus groups | Health behaviours, providing social infrastructure | Sample size: n= 1976  Area: urban  Sex: 93% female, 7% male  Age: 37% were older than 60 years | Community garden project supported by an NGO called Abalimi Bezekhaya | First author: not reported  Last author: South Africa |
| Laranjeira, R. and Laranjeira, I., 2017. Mafalala Walking Tour: Identity and Cultural Immersion at the Core of Maputo City. Museum International, 69(1-2), pp.148-155. | Southern Eastern Africa | Mozambique (Maputo) | Not applicable | Historical and archival document research, individual interviews, impromptu group interviews, focus group, participation observation, participated inventory and analysis | Providing social infrastructure, preserving cultural heritage, improving the built environment | Sample size: n= 30  Area: not reported  Sex: not reported  Age: adults | IVERCA (A community based organisation) | First author: Mozambique  Last author: Mozambique |
| Pentz, J.L., 2020. Global dance education connecting communities in Accra, Ghana and Kuwait City, Kuwait: tap (ing) to togetherness through a community collaborative program. Arts Education Policy Review, 121(3), pp.115-118 | Western Africa  Asia | Ghana (Accra)  Kuwait (Kuwait City) | Not applicable | Use of camera, videos tapes | Developing collaborations and improving family bonds | Sample size: n= not reported  Area: not reported  Sex: not reported  Age: Children ages birth to pre-kindergarten and their families | Not mentioned | First author: United States of America  Last author: No last author |
| Santos, D., Anderson, N. and Hutchinson, D., 2018. Fox Palaces: the playful occupation of a Johannesburg city park. Children's Geographies, 16(4), pp.368-379. | Southern Africa | South Africa (Gauteng Province) | Not applicable | Field notes | Empowerment of community, improving the built environment, and providing social infrastructure | Sample size: n= 40-60  Area: not reported  Sex: not reported  Age: children | University of the Free State South Africa, Humminburg Play Association | First author: South Africa  Last author: South Africa |
| Draper, C.E. and Coalter, F., 2016. “There’s just something about this club. It’s been my family.” An analysis of the experiences of youth in a South African sport-for-development programme. International Review for the Sociology of Sport, 51(1), pp.44-60. | Southern Africa | South Africa (Eastern Cape) | Not applicable | semi-structured interviews | Participation in sports and leisure activities | Sample size: n= 10  Area: not reported  Sex: 100% male  Age: 11-20 years | Laureus Sport for Good Foundation | First author: South Africa  Last author: United Kingdom |
| Wills, J., Chinemana, F. and Rudolph, M., 2010. Growing or connecting? An urban food garden in Johannesburg. Health Promotion International, 25(1), pp.33-41. | Southern Africa | South Africa (Gauteng Province) | Not applicable | Semi structured interview with themes | Health outcomes, developing collaborations, providing social infrastructure | Sample size: n= 22  Area: not reported  Sex: 100% female  Age: 40-68 years | British Council | First author: United Kingdom  Last author: South Africa |
| **Mixed-method studies** | | | | | | | | |
| Nemutandani, S., Rudolph, M., Grimsrud, A.T., De Kock, L., Lambert, E.V., Draper, C.E. and Kolbe-Alexander, T., 2010. Evaluation of a school-based physical activity intervention in Alexandra Township. South African Journal of Sports Medicine, 22(1), pp.12-19. | Southern Africa | South Africa (Johannesburg) | Case report | Structured ‚ interview, survey, questionnaire,  Field Notes | Health behaviours, health outcomes, Providing social infrastructure to improve participation and empowerment of school children | Sample size: 3262 learners, learners, 82 teachers, 7 playgrounds  Area: urban  Sex: not reported  Age: not reported | No funding reported | First author:  South Africa  Last author: South Africa |
| Ngome, I. and Foeken, D., 2012. “My garden is a great help”: gender and urban gardening in Buea, Cameroon. GeoJournal, 77(1), pp.103-118. | Western Africa | Cameroon (Fako) | Case report / Case study | Field observations | Health behaviours and improving the natural environment | Sample size: 200 individuals  Sex: 50% males, 50% females  Age: Adults | Department of Geography, University of Buea, Cameroon | First author:  Cameroon  Last author: Netherlands |
| Munien, S., Nkambule, S.S. and Buthelezi, H.Z., 2015. Conceptualisation and use of green spaces in peri-urban communities: Experiences from Inanda, KwaZulu-Natal, South Africa. African Journal for Physical, Health Education, Recreation & Dance. | Southern Africa | South Africa (KwaZulu-Natal) | Case study | Focus groups Group discussions | Improving the built environment, Improving the natural environment, empowerment of community members | Sample size: n=100 Households  Area: urban  Sex: 44% male, and 56% female  Age: Not reported | Durban Research Action Partnership for Biodiversity, Climate and People Research Group and the Environmental Protection, Climate Control Department | First author: South Africa  Last Author: South Africa |
| Clark, T.S., Friedrich, G.K., Ndlovu, M., Neilands, T.B. and McFarland, W., 2006. An adolescent-targeted HIV prevention project using African professional soccer players as role models and educators in Bulawayo, Zimbabwe. *AIDS and Behavior*, *10*(1), pp.77-83. | Southern Africa | Zimbabwe (Bulawayo) | Cohort | in depth-interviews, Focus group discussions | Health behaviours, health outcomes | Sample size: n=304 students, 155 subjects, 149 controls  Area: urban  Sex: Female: Intervention 79 (51%), Control 74 (50%)  Age: Adolescents | William and Melinda Gates Foundation. | First author: United States of America, Zimbabwe  Last Author: United States of America |
| Botcheva, L. and Huffman, L., 2004. Grassroot soccer foundation: HIV/AIDS education program: An intervention in Zimbabwe. *GRS Evaluation Report. Children’s Health Council Outcomes Research Consulting Service*. | Southern Africa | Zimbabwe (Bulawayo) | Cohort | Focus group, narrative descriptions | Health behaviours | Sample size: n=304 students  Area: urban  Sex: 53% female, 47% male  Age: adolescents | Grassroots Soccer Foundation (GRSF) and the Children’s' Health Council | First author:  Zimbabwe  Last author: United States of America |
| Abichou, H. and Zaibet, L., 2008. Evaluation of recreational nature of the Ennahli park (Tunisia). New Medit, 7(4), pp.54-60. | Northern Africa | Tunisia (Ennahli) | Cross-sectional | Structured interview , survey, questionnaire | improving the natural park environment and monetizing park goods | Sample size: n=120 park visitors  Area: urban  Sex: Not reported  Age: Children and adolescent | Not applicable | First author:  Zimbabwe  Last author: United States of America |
| Thompson, G., 2011. Reimagining surf city: Surfing and the making of the post-apartheid beach in South Africa. *The international journal of the history of sport*, *28*(15), pp.2115-2129. | Southern Africa | South Africa (Not specified) | Not specified | Not specified | Not specified | Not reported | Not reported | First author: South Africa  Last Author: Not applicable |
| Barau, A.S., Ludin, A.N.M. and Said, I., 2013. Socio-ecological systems and biodiversity conservation in African city: insights from Kano Emir’s Palace gardens. Urban ecosystems, 16(4), pp.783-800. | Western Africa | Nigeria (Kano) | Data collected from Google Earth | Observation | Improving the built environment | Not reported | Not reported | First author: Malaysia  Last Author: Malaysia |
| Freidberg, S.E., 2001. Gardening on the edge: the social conditions of unsustainability on an African urban periphery. Annals of the Association of American Geographers, 91(2), pp.349-369. | Western Africa | Burkina Faso (Bobo-Dioulasso) | Case study | Semi structured - interview , survey, questionnaire; Field Notes; Narrative descriptions; | Healthy food production; Improved income, Improved community cohesion | Sample size: n= 50 Households, 1 member of each household  Area: urban  Sex: Not reported  Age: Not reported | Fulbright Program, the National Science Foundation, and the Rocca Family Foundation for African Studies | First author:  United States of America  Last author: Not applicable |
| Diaz Olvera, L., Plat, D., Pochet, P. & Sahabana, M. Motorized two-wheelers in sub-Saharan African Cities: public and private use. 12th World Congress on Transport Research 2010 Lisbon, Portugal. | Central Africa  Western Africa | Cameroon (Douala)  Niger (Niamey) | Cross-sectional survey | In-depth interviews | Use of travel modes | Sample size: not reported  Area: urban  Sex: not reported  Age: not reported | No funding reported | First author: France  Last author: Cameroon |
| Ruysenaar, S., 2013, June. Reconsidering the ‘Letsema Principle’and the role of community gardens in food security: Evidence from Gauteng, South Africa. In Urban Forum (Vol. 24, No. 2, pp. 219-249). Springer Netherlands. | Southern Africa | South Africa (Gauteng) | Questionnaire survey | Focus group discussions, individual interview, Key informant interviews | Improving the natural environment, improving the built environment, and food security | Sample size: n= 110  Area: urban  Sex: not reported  Age: not reported | No funding reported | First author: Scotland  Last author: No last author |
| Donaldson, R., Ferreira, S., Didier, S., Rodary, E. and Swanepoel, J., 2016. Access to the urban national park in Cape Town: Where urban and natural environment meet. Habitat International, 57, pp.132-142. | Southern Africa | South Africa (Cape Town) | Questionnaire survey | Structured interview, survey | Health behaviours, natural environment, mental health, and physical infrastructure to the park | Sample size: n= 3247 individuals  Area: urban  Sex: not reported  Age: 16-24 = 9%, 25-39 = 39%, 40-65 = 44% above 65=7% | No funding reported | First author: South Africa  Last author: South Africa |
| Mistry, A. and Spocter, M., 2019. Production of Edibles and Use of Garden Waste in Domestic Gardens of a Middle-Class Suburb in Cape Town, South Africa. Journal of Urbanism: International Research on Placemaking and Urban Sustainability. | Southern Africa | South Africa (Western Cape) | Quantitative data collected from websites | person-administered surveys in the form of semi-structured interviews for qualitative data | Improving natural environment, health outcomes, and empowerment of community members | Sample size: n= 129 households  Area: urban  Sex: not reported  Age: not reported | Routledge Taylor and Francis Group; Stellenbosch University, Stellenbosch, South Africa | First author: South Africa  Last author: South Africa |
| Roberts, S. and Shackleton, C., 2018. Temporal dynamics and motivations for urban community food gardens in medium-sized towns of the Eastern Cape, South Africa. Land, 7(4), p.146. | Southern Africa | South Africa (Eastern Cape) | GIS analysis for quantitative data. | Interviews; Questionnaire administration; physical observation by researchers for qualitative | Examining the benefits and challenges experienced by community gardeners | Sample size: n= 69 individuals  Area: urban  Sex: 51% Male; 49% Female  Age: 18-56 years | Rhodes University, Grahams town 6140, South Africa; Sky Roberts; Charlie Shackleton; South African Research Chairs Initiative of the Department of Science and Technology and the National Research Foundation of South Africa | First author: South Africa  Last author: South Africa |
| Hershow, R.B., Gannett, K., Merrill, J., Kaufman, E.B., Barkley, C., DeCelles, J. and Harrison, A., 2015. Using soccer to build confidence and increase HCT uptake among adolescent girls: a mixed-methods study of an HIV prevention programme in South Africa. Sport in society, 18(8), pp.1009-1022. | Southern Africa | South Africa (Cape Town) | Use of questionnaire for quantitative and focus group discussion for qualitative. | Sample size: n= 514 participants  Area: urban  Sex: 100% female  Age: 12-16 years | Health behaviours, and health outcomes | Sample size: n= 3262 learners  Area: urban  Sex: 100% females  Age: 12-16 years | The Elton John AIDS Foundation (EJAF), MAC AIDS Fund; USAID-New Partners Initiative, USAID-Global Development Alliance (GDA) | First author: South Africa  Last author: South Africa |
| **Quantitative studies** | | | | | | | | |
| Green, C.A., Mans, G.G., Spocter, M. and McKelly, D., 2010. Evaluation of community social facilities and recreational space in City of Cape Town: current and future provision for 2016 and optimal location of new facilities. *CSIR, CSIR/BE/PSS/ER/2010/0041/B*, pp.1-8. | Southern Africa | South Africa (Cape town) | Case-control | Not Applicable | Recreation | Sample size: n=256 individuals  Area: urban  Sex: Not reported  Age: Not reported | Not reported | First author: South Africa  Last Author: South Africa |
| Caldwell, L.L., Younker, A.S., Wegner, L., Patrick, M.E., Vergnani, T., Smith, E.A. and Flisher, A.J., 2008. Understanding leisure-related program effects by using process data in the HealthWise South Africa project. Journal of park and recreation administration, 26(2), p.146. | Southern Africa | South Africa (Cape town) | Cohort study | Not applicable | Recreation, sports | Sample size: n=Not reported  Area: urban  Sex: Not reported  Age: Not reported | National Institute of Drug Abuse | First author: United States of America  Last Author: Norway |
| Fuller, C.W., Junge, A., Dorasami, C., DeCelles, J. and Dvorak, J., 2011. ‘11 for Health’, a football-based health education programme for children: a two-cohort study in Mauritius and Zimbabwe. British journal of sports medicine, 45(8), pp.612-618. | Eastern and Southern Africa | Mauritius, Zimbabwe | Cohort | Not applicable | health outcomes, healthy behaviours, improve quality of life. | Sample size: n=389nMauritius, Zimbabwe 395  Area: urban  Sex: 50% males, 50% female  Age: Adolescents | Fédération Internationale de Football Association | First author: Switzerland  Last Author: Switzerland |
| Khan, N. and Hendrin, M., 2010. Using football for HIV/AIDS prevention in Africa. Football for an HIV-Free Generation. | Sub-Saharan Africa | South Africa, Zimbabwe, Zambia, Lesotho, Kenya, Namibia, Senegal, Ghana, Cameroon, Malawi, Botswana, Uganda | Case reports | Not applicable | Health behaviours | Sample size: Not reported  Area: Not reported  Sex: Not reported  Age: Not reported | Not mentioned | First author: Not reported  Last Author: Not reported |
| Uys, M., Draper, C.E., Hendricks, S., de Villiers, A., Fourie, J., Steyn, N.P. and Lambert, E.V., 2016. Impact of a South African school-based intervention, HealthKick, on fitness correlates. American journal of health behavior, 40(1), pp.55-66. | Southern Africa | South Africa | Cohort | Not applicable | Health behaviours, Health outcomes, improve participation | Sample size: 16 schools  Area: Not reported  Sex: Not reported  Age: Not reported | World Diabetes Foundation | First author: South Africa  Last Author: South Africa |
| Beintema, A.J., 1991. Management of the Djoudj National Park in Senegal. Landscape and Urban Planning, 20(1-3), pp.81-84. | Western Africa | Senegal (Northern Biffeche) | Case study | Not applicable | Not reported | Sample size: Not reported  Area: Not reported  Sex: Not reported  Age: Not reported | Not reported | First author: Netherlands  Last Author: Not applicable |
| Simelane, T.S., Kerley, G.I.H. and Knight, M.H., 2006. Reflections on the relationships between communities and conservation areas of South Africa: The case of five South African national parks. *Koedoe*, *49*(2), pp.85-102 | Southern Africa | South Africa (Eastern Cape) | Cross-sectional survey | Not applicable | Improving the natural environment, Providing social infrastructure to improve participation and empowerment of community members | Sample size: 2000 people  Area: rural  Sex: Not reported  Age: Adolescents and adults | South African National Parks | First author: South Africa  Last Author: South Africa |
| Gunter, A., 2011. Stadium upgrades as local economic development: the fallacy of the Ellis Park Sports Precinct upgrade as LED. *South African Geographical Journal*, *93*(1), pp.75-88. | Southern Africa | South Africa (Johannesburg) | Case report | Not applicable | Improving the built environment, Providing social infrastructure to improve participation and empowerment of community members, economic development | Sample size: n= 139  Area: urban  Sex: not reported  Age: not reported | Department of Geography and Environmental Management (University of Johannesburg) | First author: South Africa  Last author: Not last author |
| Mwakiwa, E., Maparara, T., Tatsvarei, S. and Muzamhindo, N., 2018. Is community management of resources by urban households, feasible? Lessons from community gardens in Gweru, Zimbabwe. *Urban Forestry & Urban Greening*, *34*, pp.97-104. | Southern Africa | Zimbabwe (Gweru) | Secondary data sources, and key informant and household interviews. | Not applicable | Determine the factors that affected public participation in community gardening | Sample size: n= 140 households  Area: urban  Sex: not reported  Age: not reported | Not mentioned | First author: Zimbabwe  Last author: Zimbabwe |
| Motamedi, M., Caldwell, L.L., Weybright, E.H., Jones, D., Wegner, L. and Smith, E.A., 2020. Doing a leisure activity because there is nothing else to do: Related outcomes and intervention effects for adolescents. *Journal of Leisure Research*, *51*(1), pp.1-15. | Southern Africa | South Africa (Western Africa) | Youth self-report surveys | Not applicable | Health behaviours and health Outcomes | Sample size: n= 6243 individuals  Area: not reported  Sex: 52% female; 48% male  Age: students, mean age=14 | National Institute of Drug Abuse, Institute of Education Sciences | First author: United States of America  Last author: South Africa |
| Tegen, H., Chanie, Y. and Jembere, M., 2015. Establishment of Model Home Garden for Home Consumption and Income Generation of Poor and HIV Victim Women at Bahir Dar, Ethiopia. Journal of Agricultural Science and Technology A, 5(3), pp.183-189. | Eastern Africa | Ethiopia (Amhara state) | Household survey and observation | Not applicable | Developing collaborations, improving the natural environment, providing social infrastructure, community empowerment, and health outcomes | Sample size: n= 5 households  Area: urban  Sex: 100% female  Age: women | Amhara Regional Agricultural Research Institute (ARARI) and Rural Capacity Building Project | First author: Ethiopia  Last author: Ethiopia |
| Caldwell, L. L., Patrick, M. E., Smith, E. A., Palen, L.-A. & Wegner, L. 2010. Influencing adolescent leisure motivation: Intervention effects of health wise South Africa. Journal of Leisure Research, 42, 203-220. | Southern Africa | South Africa (Western Cape) | Completion of FTMS-A by adolescents | Not applicable | Assessment of motivation levels which may affect health behaviours | Sample size: n= 2193 students  Area: urban  Sex: 51% female, while 49% male  Age: mean age= 14 years | National Institute of Health | First author: United States, South Africa, Malaysia  Last author: South Africa |
| Kolbe-Alexander, T., Lambert, E.V. and Charlton, K.E., 2006. Effectiveness of a community basedlow intensity exercise program for older adults. *The Journal of nutrition, health & aging*, *10*(1), p.21. | Southern Africa | South Africa (Cape Town) | A questionnaire, the Yale Physical Activity Survey (YPAS) was administered | Not applicable | Health behaviours, health outcomes, developing collaborations, providing social infrastructure to improve participation and empowerment of community members | Sample size: n= not reported  Area: not reported  Sex: not reported  Age: not reported | MRC/UCT Research Unit for Exercise Science and Sports Medicine, Department of Medicine, University of Cape Town and Chronic Diseases of Lifestyle Unit, South Africa | First author: South Africa  Last author: South Africa |
| Rotheram-Borus, M.J., Tomlinson, M., Durkin, A., Baird, K., DeCelles, J. and Swendeman, D., 2016. Feasibility of using soccer and job training to prevent drug abuse and HIV. AIDS and Behavior, 20(9), pp.1841-1850. | Southern Africa | South Africa (Western Cape) | Questionnaires | Not applicable | Health behaviours, health outcomes, providing social infrastructure to improve participation and empowerment of community member | Sample size: n= 142 individuals  Area: urban  Sex: 100% male  Age: 18-25 years, and mean age =21.9 | National Institute on Drug Abuse (NIDA), Centre  for HIV Identification, Prevention, and Treatment (CHIPTS) NIMH; the UCLA Centre for AIDS Research (CFAR); the National Centre for Advancing Translational Sciences through UCLA CSTI; the William T. Grant Foundation; and the National Research Foundation | First author: United States of America  Last author: United States of America |
