# Supplementary figures and images for: Urban public space initiatives and health in Africa: A mixed-methods systematic review
Source: PLOS Glob Public Health. 2024 Oct 15;4(10):e0003709. doi: 10.1371/journal.pgph.0003709 (PMC11478912; doi:10.1371/journal.pgph.0003709)

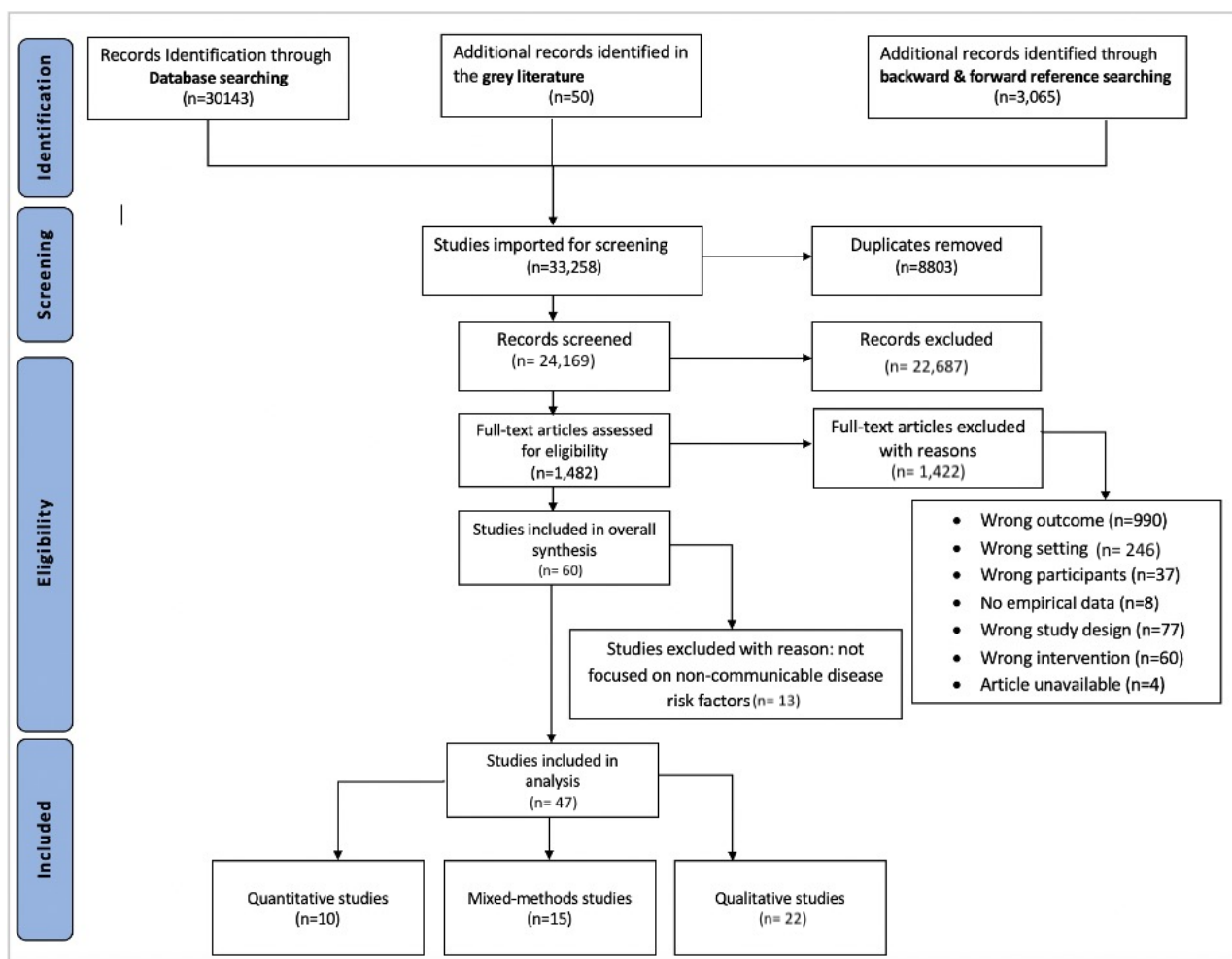

Supplement: S1 Fig — (PDF) [file pgph.0003709.s015.pdf]

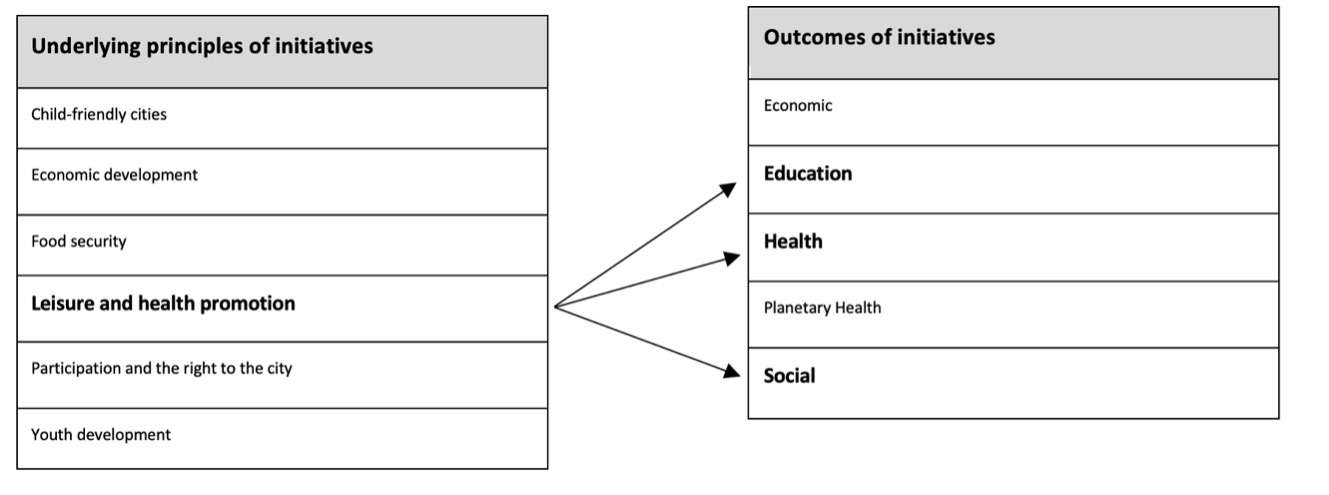

Supplement: S2 Fig — (TIF) [file pgph.0003709.s016.tif]
