# Supplementary material for: Urban public space initiatives and health in Africa: A mixed-methods systematic review
Source: PLOS Glob Public Health. 2024 Oct 15;4(10):e0003709. doi: 10.1371/journal.pgph.0003709 (PMC11478912; doi:10.1371/journal.pgph.0003709)
Supplement: Attachment — Submitted filename: Response to Reviewers_ 06 Mar.docx [file pgph.0003709.s017.docx]

06 March 2024

Dear Editor,

Many thanks for the very constructive comments you have sent us. In essence, the reviewer has three main comments: (a) considering that the findings are extracted from a majority of South African papers, (b) restructure and shorten the discussion section and (c) double check the spelling in the manuscript. In addition, we have also reviewed the manuscript to make a stronger case for why studying African public space initiatives across the whole continent can benefit scholarship and research on this topic. Our detailed response to the journal requirements and the reviewers comments are provided below in the table.

|  | ***Journal requirements*** | ***Our response*** |
| --- | --- | --- |
| 1 | Please provide separate figure files in .tif or .eps format only and remove any figures embedded in your manuscript file. Please also ensure all files are under our size limit of 10MB. | We have provided 2 figures as separate files. |
| 2 | We have noticed that you have uploaded Supporting Information files, but you have not included a list of legends. Please add a full list of legends for your Supporting Information files after the references list. | We have added a list of legends for the supplementary files. |
| 3 | In the online submission form, you indicated that &quot;The data that was extracted from available published and peer-reviewed studies that support the findings of this study are available from the corresponding author, [MT], upon reasonable request&quot;. All PLOS journals now require all data underlying the findings described in their manuscript to be freely available to other researchers, either 1. In a public repository, 2. Within the manuscript itself, or 3. Uploaded as supplementary information. | We have now uploaded the data extracted from the studies as a supplementary file entitled ‘Characteristics of included studies’. |
|  | ***Reviewer’s comments*** | ***Our response*** |
| 1 | I would like to commend the authors for conducting such a thorough review. | Thank you very much. |
| 2 | I understand that a lot of global health work focuses on Africa broadly, but the continent is vast a very diverse. The authors should make a stronger case for why studying african public space initiatives as a whole, as opposed to say, sub-regions with similar climate and climate-change related challenge, or say, a subset of countries with similar socioeconomics, as they may be similar in terms of advances in urban and rural infrastructure. | Modification in text (line 97-106) |
| 3 | As the authors note, the vast majority of papers they identified are from South Africa. Both in terms of climate (types of seasons etc) and socioeconomics, South Africa is closer to many Western countries than many of its African counterparts. Perhaps it makes more sense to split this paper. Otherwise, can you apply to sub-saharan/central/west African countries the insights gleaned from a number of papers with a vast majority about South Africa? | For the reasons mentioned in response to comment 2, we value the comprehensive approach adopted in the paper to investigate and articulate this review across all countries of the continent. We therefore prefer not to split the paper into two. We have added further clarification on this matter in lines 508-511. |
| 4 | Double check spelling/numbers. in section 3.2, the number after school is &ldquo;x.&rdquo; | Checks on spelling have been undertaken in the entire manuscript and numbering has been modified as from line 298 onwards. |
| 5 | I appreciate the robustness of the methods, and the thoroughness with which the authors wrote the results section. | Thank you. |
| 6 | I suggest that the authors limit the discussion to:  1. hilighliting key findings 2. What do findings uniquely add to the literature? 3. explain/theorize about your findings  4. put findings in concert with existing literature, in this case, perhaps work in this area based on other regions of the world. 5. Highlight gaps in the literature, growth areas, future directions. 6. Highlight study limitations | We have shortened the discussion section and restructured based on proposed sections. |
| 7 | In addition to community cohesion and physical activity, I recommend that authors discuss nuances between different types of nature public spaces, and their respective benefits to the environment especially in the context of climate change and also how such public spaces can serve as an opportunity for simple relaxation, which in itself can be good for health. | We have rephrased some sentences around this in the discussion section (see line 410). |
| 8 | The recommendations to governments, while very good, belong in a separate policy brief or perspective. I think they go beyond the stated goal/scope of the manuscript. | We have removed the recommendations to governments. |

We hope that the revised paper now meets your standards.

With best wishes,

Meelan Thondoo and co-authors
