# Supplementary material for: Urban public space initiatives and health in Africa: A mixed-methods systematic review
Source: PLOS Glob Public Health. 2024 Oct 15;4(10):e0003709. doi: 10.1371/journal.pgph.0003709 (PMC11478912; doi:10.1371/journal.pgph.0003709)
Supplement: Attachment — Submitted filename: Lettertotheeditor_2Jul.docx [file pgph.0003709.s018.docx]

Cambridge, 02 July

Dear Editor,

We are resubmitting our paper titled ‘Urban Public Space Initiatives and Health in Africa: A Mixed-Methods Systematic Review’ for your consideration.

We consider this article, especially in its current form to be a vital piece on public space initiatives (PSI) in African contexts. This will allow for the identification of synergies with stakeholders across different sectors to act on health in cities.

We have reworked the abstract and refined the introduction to make the reasoning and approach behind our paper more obvious to the reader. In addition to our submission, we have included a response to the reviewers’ comments to demonstrate how we have incorporated the feedback received to strengthen our resubmission.

Thank you for your consideration and we look forward to hearing from you.

Sincerely yours,
Dr. Thondoo and co-authors

| Reviewer 2 comments | Response and modifications |
| --- | --- |
| I see some good value in publishing such works; thus, I am mostly positive about their publication. I only have a few minor points, which might be better clarified. Based on the existing studies, it provides a good overview of public space initiatives (PSI) in African contexts. The method is clear, the results are reasonable, and the study is well-written. | Thank you very much. |
| The abstract is too brief to say anything clearly. I am not sure if it is a journal requirement. But if it is not, then I suggest expanding the abstract a bit more to give further information to the reader, the background of the paper, and some additional info about the PSI-related activity type and settings. | The abstract has been rewritten. |
| The intro could give a bit more information on PSI interventions from a theoretical viewpoint, such as why and what types are common in contexts of Africa. I missed some points about the PSI in the intro; after reading the result, I got the idea clearly, but the authors could add a few more lines about the PSI in the intro, such as Activity types and Underlying principles. | Theoretical explanation on PSI has been added in the introduction. |
| As a result, table 2 can be converted into charts and maps that would be easy to follow. | Thank you for the suggestion to convert Table 2 into charts and maps. After careful consideration, we have decided to keep the data in a table format as it provides a comprehensive and detailed overview of the information, which aligns with our analytical approach for this paper.  The results are very uneven and do not lend themselves to clear or illustrative visualizations in maps or charts. For example, most countries were represented with 1 study (n=1) compared to South Africa which was covered by many more (n=30). This disparity would create a visual representation that might be misleading or unclear in a map format. |
| Other parts of the paper are quite clear, and I do not have further comments. | Thank you. |
